# The Fungal Microbiome Is an Important Component of Vineyard Ecosystems and Correlates with Regional Distinctiveness of Wine

**DOI:** 10.1128/mSphere.00534-20

**Published:** 2020-08-12

**Authors:** Di Liu, Qinglin Chen, Pangzhen Zhang, Deli Chen, Kate S. Howell

**Affiliations:** a School of Agriculture and Food, Faculty of Veterinary and Agricultural Sciences, University of Melbourne, Parkville, Australia; University of Georgia

**Keywords:** wine regionality, microbial biogeography, fungal diversity, climate, soil, soil microbiology, yeasts

## Abstract

The flavors of fermented plant foods and beverages are formed by microorganisms, and in the case of wine, the location and environmental features of the vineyard site also imprint the wine with distinctive aromas and flavors. Microbial growth and metabolism play an integral role in wine production, by influencing grapevine health, wine fermentation, and the flavor, aroma, and quality of finished wines. The contributions by which microbial distribution patterns drive wine metabolites are unclear, and while flavor has been correlated with fungal and bacterial composition for wine, bacterial activity provides fewer sensorially active biochemical conversions than fungi in wine fermentation. Here, we collected samples across six distinct wine-growing areas in southern Australia to investigate regional distribution patterns of fungi and bacteria and the association with wine chemical composition. Results show that both soil and must microbiota distinguish wine-growing regions. We found a relationship between microbial and wine metabolic profiles under different environmental conditions, in particular measures of soil properties and weather. Fungal communities are associated with wine regional distinctiveness. We found that the soil microbiome is a source of grape- and must-associated fungi and suggest that weather and soil could influence wine characteristics via the soil fungal community. Our report describes a comprehensive scenario of wine microbial biogeography where microbial diversity responds to the surrounding environment and correlates with wine composition and regional characteristics. These findings provide perspectives for thoughtful human practices to optimize food composition through understanding fungal activity and abundance.

**IMPORTANCE** The composition of soil has long been thought to provide wine with characteristic regional flavors. Here, we show that for vineyards in southern Australia, the soil fungal communities are of primary importance for the aromas found in wines. We propose a mechanism by which fungi can move from the soil through the vine.

## Abstract (precis)

The composition of soil has long been thought to provide wine with characteristic regional flavors. Here, we show that for vineyards in southern Australia, the soil fungal communities are of primary importance for the aromas found in wines. We propose a mechanism by which fungi can move from the soil through the vine.

## INTRODUCTION

Regional distinctiveness of wine traits, collectively known as “*terroir*,” can be measured by chemical composition and sensory attributes (1–3), and this variation has been related to the physiological responses of grapevines to local environments, such as soil properties (e.g., soil type, texture, and nutrient availability), climate (temperature, precipitation, and solar radiation), topography, and human-driven agricultural practices (4–6). Wines made from the same grape cultivar but grown in different regions are appreciated for their regional diversity, increasing price premiums and market demand (5). However, the vineyard and winery factors that drive regional wine quality traits remain elusive.

Microorganisms, including yeasts, filamentous fungi, and bacteria, originate in the vineyard, are impacted upon by the built environment (winery), and play a decisive role in wine production and quality of the final wine (7–9). The fermentative conversion of grape must (or juice) into wine is a complex and dynamic process, involving numerous transformations by multiple microbial species (10). The majority of fermentations involve *Saccharomyces* yeasts conducting alcoholic fermentation (AF) and lactic acid bacteria (LAB) for malolactic fermentation (MLF), but many other species are present and impact the chemical composition of the resultant wine (11, 12). Recent studies propose the existence of nonrandom geographical patterns of microbiota in grapes and wines (13–19). Few studies have explored the associations between microbial communities and wine chemical composition (20, 21). Bokulich et al. (20) suggested that wine metabolites correlated with the bacterial and fungal consortia. There was a weaker correlation for fungi than bacteria with the metabolic profiles in finished Cabernet Sauvignon wines which was attributed to bacterial bioconversions during MLF. Knight et al. (21) showed empirically that regionally distinct Saccharomyces cerevisiae populations drove metabolic distinctiveness in the resultant wines, but S. cerevisiae is just one fungal species associated with winemaking. The diverse taxonomy and biochemical diversity of fungi in general are known to make important contributions to plant health and function, but their occurrence and impact beyond *Saccharomyces* spp. have not been comprehensively investigated in soil, grapes, and vines. Learning whether the fungi present in vineyard ecosystems correspond to and impact upon wine production could give valuable information about how vine health and wine flavor are linked.

The composition and structure of vineyard soil have long been believed to be of great importance in determining wine characteristics and flavor. Vineyard soil provides the grapevine with water and nutrients, and soil type and properties profoundly affect vine growth and development (5). Soil-borne microbiota associates with grapevines in a beneficial, commensal, or pathogenic way and determines soil quality and host growth and health. For example, soil microbes can mineralize soil organic matter and trigger plant defense mechanisms and thus influence the flavor and quality of grapes and final wines (22, 23). Alternatively, soil was previously suggested to be a potential source reservoir of grapevine-associated microbiota (15, 24) and some of soil microbes can influence fermentation and contribute to final wine characteristics (8, 24). Overall, biogeographic boundaries can constrain the vineyard soil microbiota (23, 25–28), but correlations between soil microbiota and wine attributes are weak (15).

Limited but increasing evidence reveals that environmental heterogeneity conditions microbial biogeography in wine production on different spatial scales (recently reviewed by Liu et al. [29]) (13, 24, 26, 28, 30, 31). Local climatic conditions significantly correlate with microbial compositions in grape musts; for example, precipitation and temperature have been found to correlate with the abundance of filamentous fungi (for example, *Cladosporium* and *Penicillium* spp.) and of ubiquitous bacteria (for example, members of the *Enterobacteriaceae* family) (13), as well as of yeast populations (particularly *Hanseniaspora* and *Metschnikowia* spp.) (30). Dispersal of soil microbiota is driven by soil physicochemical properties such as soil texture, soil pH, and carbon (C) and nitrogen (N) pools (24, 26, 27), with some influences from topological characteristics (for example, orientation of the vineyard) (26, 32). Soil microbiome/bacteria may colonize grapes by physical contact (being moved by rain splashes, dust, and winds) (24) or by migration through the plant (xylem/phloem) from the rhizosphere to the phyllosphere (33). Insects help the movement and dispersal of microbes in the vineyard and winery ecosystem; for example, honeybees, social wasps, and drosophilid flies can vector yeasts among different microhabitats (34–36). Vineyard microbes enter the winery in association with grapes or must, so the effects of environmental conditions are finally reflected on microbial consortia in wine fermentation. How environmental conditions modulate microbial ecology from the vineyard to the winery and shape regional distinctiveness of wine is still largely unknown.

Here, we initially tested microbial contribution to wine regional characteristics. To tackle this issue, we sampled microbial communities from the vineyard to the winery across six geographically separated wine-producing regions in southern Australia. We evaluated the volatile chemicals of wines made with Pinot Noir grapes to validate the hypothesis that these different regions have differently flavored wines. Using next-generation sequencing (NGS) to profile bacterial and fungal communities, we demonstrate that the soil and must microbiota exhibit distinctive regional patterns and that this correlates to the wine metabolome. Associations among soil and wine microbiome, abiotic factors (weather and soil properties), and wine regionality were modeled by random forest and structural equation modeling (SEM), highlighting the important contributions of fungal communities. We then tested a potential route of transmission of wine-related fungi from the soil to the grapes by isolating yeasts from the xylem/phloem of grapevines to further explore the role of fungi in wine regionality. Using vineyards, grapes, and wine as a model food system, we have related the regional identity of an agricultural commodity to biotic components in the growing system to show the importance of conserving regional microbial diversity to produce distinctive foods and beverages.

## RESULTS

### Chemical composition/aroma profiles separate wines based on geographic location.

**FIG 1:**
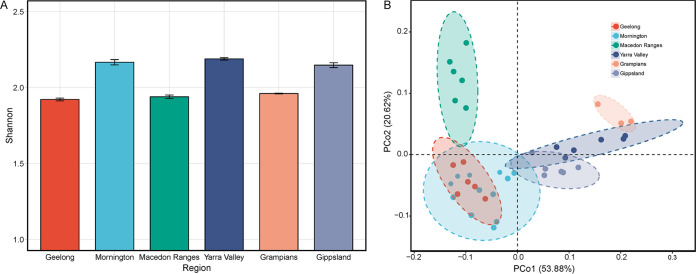
Wine metabolome shows regional variation across six wine-growing regions in 2017. Shown are α-diversity (Shannon index) (A) and PCoA (B) based on Bray-Curtis dissimilarity obtained from comparing volatile profiles.

Using headspace solid-phase microextraction gas-chromatographic mass-spectrometry (HS-SPME–GC-MS), we analyzed the volatile compounds of Pinot Noir wine samples (MLF-End) to represent wine metabolite profiles coming from different growing regions and compared the results directly to the microbial communities inhabiting the musts from which these wines were fermented. In all, 88 volatile compounds were identified in these wines, containing 48 regionally differential compounds (see Table S2 in the supplemental material). Here, we used α- and β-diversity measures to further elucidate wine complexity and regionality, respectively. In wines of 2017 vintage, α-diversity varied with regional origins (analysis of variance [ANOVA], F = 36.021, *P* < 0.001), with higher Shannon indices observed for the wines from regions of Mornington, Yarra Valley, and Gippsland (*H* = 2.17 ± 0.05) than for those from other regions (*H* = 1.94 ± 0.03) (Fig. 1A). Overall, wine aroma profiles displayed significant regional differentiation across both vintages based on Bray-Curtis dissimilarity (permutational multivariate analysis of variance [PERMANOVA], coefficient of determination [*R*^2^] = 0.566, *P* < 0.001) and the clustering patterns became more distinct and the *R*^2^ values improved in comparisons of regional differences in wines of 2017 vintage (PERMANOVA; *R*^2^ = 0.703, *P* < 0.001) (Table S3). Principal-coordinate analysis (PCoA) showed that 74.5% of the variance was explained by the first two principal coordinates in 2017, and on PCo1 there were some wines within regions grouped together (Fig. 1B).

10.1128/mSphere.00534-20.7 TABLE S2 ANOVA results of analyses of wine volatile compounds among wine-growing regions in 2017. Download Table S2, XLSX file, 0.02 MB. Copyright © 2020 Liu et al. 2020 Liu et al. This content is distributed under the terms of the Creative Commons Attribution 4.0 International license.

10.1128/mSphere.00534-20.8 TABLE S3 Permutational multivariate analysis of variance (PERMANOVA) performed using distance matrices for categories of effects on microbial and wine aroma diversity. Download Table S3, XLSX file, 0.01 MB. Copyright © 2020 Liu et al. 2020 Liu et al. This content is distributed under the terms of the Creative Commons Attribution 4.0 International license.

### Microbial ecology from the vineyard to the winery.

To test the role of microbial diversity in regional traits of wine from the vineyard to the winery, 150 samples covering soils, musts, and fermentations were collected to analyze wine-related microbiota. A total of 11,508,480 16S rRNA and 12,403,610 internal transcribed spacer (ITS) high-quality sequences were generated from the samples, which were clustered into 13,689 bacterial and 8,373 fungal operational taxonomic units (OTUs) with a threshold of 97% pairwise identity.

**FIG 2:**
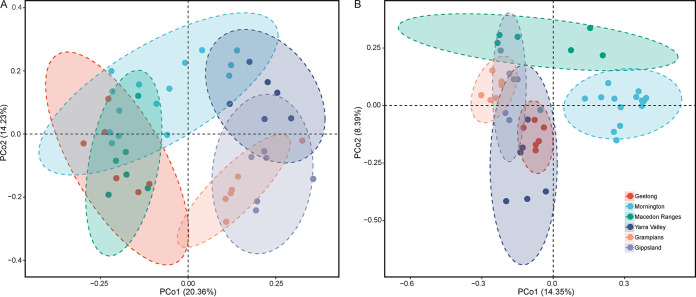
Regional patterns of vineyard soil microbial communities demonstrated by Bray-Curtis distance PCoA of bacterial communities (A) and fungal communities (B).

The dominant bacterial taxa across all soil samples were *Actinobacteria*, *Proteobacteria*, *Acidobacteria*, *Chloroflexi*, *Verrucomicrobia*, *Bacteroidetes*, *Gemmatimonadetes*, *Firmicutes*, *Planctomycetes*, and *Nitrospirae* (see Fig. S2A in the supplemental material). Compared with bacteria, soil fungal communities were less diverse (Table S4). *Ascomycota* was the most abundant and diverse phylum of fungi, accounting for 72% of reads, followed by *Basidiomycota*, *Mortierellomycota*, *Chytridiomycota*, and *Olpidiomycota* (Fig. S2B). The microbial diversity (α-diversity, Shannon index) differed significantly between regions for both bacteria and fungi (ANOVA; *F*_bacteria_ = 4.645, *P* < 0.01; *F*_fungi_ = 4.913, *P* < 0.01). Soil microbial communities varied widely across different grape-growing regions, and significant differences were observed in both bacterial taxonomic dissimilarity and fungal taxonomic dissimilarity based on Bray-Curtis distances matrices at the OTU level (PERMANOVA; *R*^2^_bacteria_ = 0.318, *P* < 0.001; *R*^2^_fungi_ = 0.254, *P* < 0.001), with clearer differences within a single vintage (PERMANOVA; *R*^2^_bacteria 2017_ = 0.392, *P* < 0.001; *R*^2^_fungi 2017_ = 0.419, *P* < 0.001) (Table S3). In 2017, soil samples from the different growing regions (except Yarra Valley and Gippsland) were able to be discriminated based on fungal communities (Fig. 2B), whereas regional separation of bacteria was weaker, with overlap of regions (Fig. 2A).

10.1128/mSphere.00534-20.3 FIG S2 Vineyard soil microbial community compositions across all samples from six grape-growing regions. Shown are average percentages of taxa (characterized to the phylum level) across sites in each region. (A) Dominant bacterial phyla with greater than 1.0% relative abundance. (B) Fungal phyla. Download FIG S2, TIF file, 1.0 MB. Copyright © 2020 Liu et al. 2020 Liu et al. This content is distributed under the terms of the Creative Commons Attribution 4.0 International license.

10.1128/mSphere.00534-20.9 TABLE S4 α-Diversity (Shannon index) data representing soil and must microbial communities from six wine-growing regions in 2017 and 2018. Download Table S4, XLSX file, 0.01 MB. Copyright © 2020 Liu et al. 2020 Liu et al. This content is distributed under the terms of the Creative Commons Attribution 4.0 International license.

**FIG 3:**
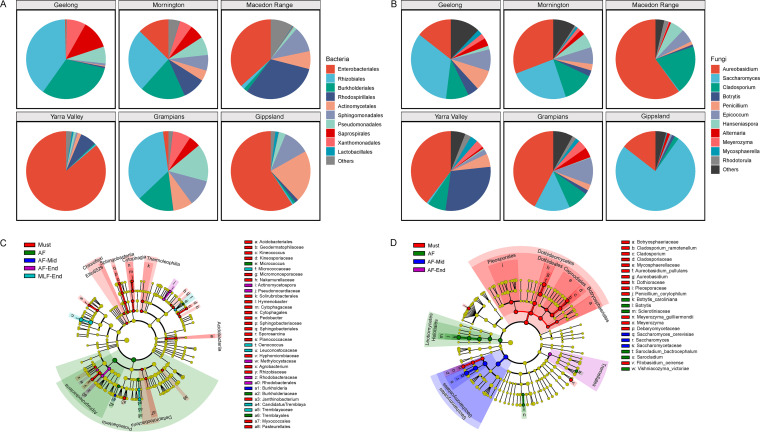
Microbiota exhibit regional differentiation in musts for both bacterial and fungal profiles. The stage of fermentation influences microbial composition of bacteria and fungi. (A) Must bacterial taxa with greater than 1.0% relative abundance at the order level, and *Lactobacillales* (average abundance, 0.4%). (B) Must fungal taxa with greater than 1.0% relative abundance at the genus level. (C and D) Linear discriminant analysis (LDA) effect size (LEfSe) taxonomic cladograms comparing all musts and wines categorized by fermentation stage. Significantly discriminant taxon nodes (C, bacteria; D, fungi) are colored and branch areas are shaded according to the highest-ranked stage for that taxon. For each taxon detected, the corresponding node in the taxonomic cladogram is colored according to the highest-ranked group for that taxon. If the taxon is not significantly differentially represented between sample groups, the corresponding node is colored yellow.

Among grape musts, bacterial communities of both vintages across six wine-growing regions consisted of the ubiquitous bacteria *Enterobacteriales*, *Rhizobiales*, *Burkholderiales*, *Rhodospirillales*, *Actinomycetales*, *Sphingomonadales*, *Pseudomonadales*, *Saprospirales*, and *Xanthomonadales*, which do not participate in wine fermentations or spoilage (7). Members of the LAB *Lactobacillales*, responsible for malolactic fermentation, were present in low abundance (0.4% on average) in the must (Fig. 3A). Fungal profiles were dominated by filamentous fungi, mostly of the genera *Aureobasidium*, *Cladosporium*, *Botrytis*, *Epicoccum*, *Penicillium*, *Alternaria*, and *Mycosphaerella*, with notable populations of yeasts, including *Saccharomyces*, *Hanseniaspora*, and *Meyerozyma*, as well as the Basidiomycota genus *Rhodotorula* (Fig. 3B). Pinot Noir musts exhibited significant regional patterns for fungal communities across vintages 2017 and 2018 based on Bray-Curtis dissimilarity at the OTU level (PERMANOVA; *R*^2^_fungi_ = 0.292, *P* < 0.001) but no significant differences for bacterial communities across both vintages (PERMANOVA; *R*^2^_bacteria_ = 0.108, *P* = 0.152) (Table S3), as well as regarding community diversity (ANOVA; *F*_bacteria_ = 1.567, *P* = 0.374; *F*_fungi_ = 5.142, *P* < 0.01) (Table S4). Within the 2017 vintage, both bacteria and fungi in the must showed distinctive compositions on the basis of region (PERMANOVA; *R*^2^_bacteria_ = 0.342, *P* < 0.001; *R*^2^_fungi_ = 0.565, *P* < 0.001) (Fig. 3A and B) (Table S3), with a more distinct trend and improved *R*^2^ coefficient values for fungi. Notably, the relative abundances of Saccharomyces yeasts between regions ranged widely from 1.3% (Macedon Range) to 65.6% (Gippsland) (Fig. 3B). As the wine fermentation proceeded, fermentative populations, including yeasts and LAB, grew and dominated, thus reshaping the community diversity (Fig. S3A and B) and composition (Fig. S3C and D). Fungal species diversity collapsed as alcoholic fermentation progressed (ANOVA; *F* = 6.724, *P* < 0.01) (Fig. S3B), while the impact of the fermentation on bacterial diversity was insignificant (ANOVA; *F* = 1.307, *P* = 0.301), with a slight decrease at early stages and recovery at the end of fermentation (Fig. S3A). Linear discriminant analysis (LDA) effect size (LEfSe) determinations further identified differentially abundant taxa (Kruskal-Wallis rank sum test, α < 0.05) associated with fermentation stages (Fig. 3). For fungal populations, *Dothideomycetes* (including *Aureobasidium* and *Cladosporium*), *Debaryomycetaceae* (notably, yeast Meyerozyma guilliermondii), Penicillium corylophilum, and Filobasidium oeirense were significantly abundant in the grape must, including *Leotiomycetes*, *Sarocladium*, and Vishniacozyma victoriae in early fermentations (AF), *Saccharomycetes* yeasts (notably S. cerevisiae) in mid-fermentations (AF-Mid), and *Tremellales* (notably yeast Vishniacozyma victoriae) at the end of fermentation (AF-End) (Fig. 3D). For bacterial communities, *Acidobacteriia* (spoilage), *Chloroflexi*, Deltaproteobacteria, *Sphingobacteriia*, *Cytophagia*, *Planococcaceae*, and *Rhizobiaceae* were observed with higher abundances in the must; *Proteobacteria* (including *Burkholderiaceae* and *Tremblayales*, spoilage) and *Micrococcus* in the AF; *Burkholderia* spp. in the AF-Mid; *Rhodobacterales* and *Pseudonocardiaceae* in the AF-End; and LAB *Leuconostocaceae* (notably *Oenococcus*) in the MLF-End (Fig. 3C). Regional differences in microbial profiles were not significant in the finished wines (PERMANOVA; *R*^2^_bacteria_ = 0.149, *P* = 0.321; *R*^2^_fungi_ = 0.109, *P* = 0.205) (Table S3).

10.1128/mSphere.00534-20.4 FIG S3 The stage of fermentation influences microbial diversities. (A and B) Bacterial (A) and fungal (B) α-diversity (Shannon index) changes during wine fermentation. (C and D) Bray-Curtis distance PCoA of bacterial communities (C) and fungal communities (D) according to the fermentation stage. Download FIG S3, TIF file, 1.7 MB. Copyright © 2020 Liu et al. 2020 Liu et al. This content is distributed under the terms of the Creative Commons Attribution 4.0 International license.

To uncover the impact of growing season (vintage) on wine regionality and related microbiota, five vineyards in Mornington were sampled in 2017 and 2018 to perform comparisons within and between vintages. Within these five vineyards alone, both microbial communities and wine aroma showed a significant influence from vintage effects (Table S3). In large-scale comparisons of all samples, vintage only weakly impacted microbial and wine aroma profiles; in particular, an insignificant influence on fungi was seen (PERMANOVA; *R*^2^_fungi_ = 0.049, *P* = 0.066) (Table S3). We used 2017 vintage data to further explore microbial biogeography and wine regionality in the following analyses.

### Multiple factors modify wine regionality in the vineyard.

**FIG 4:**
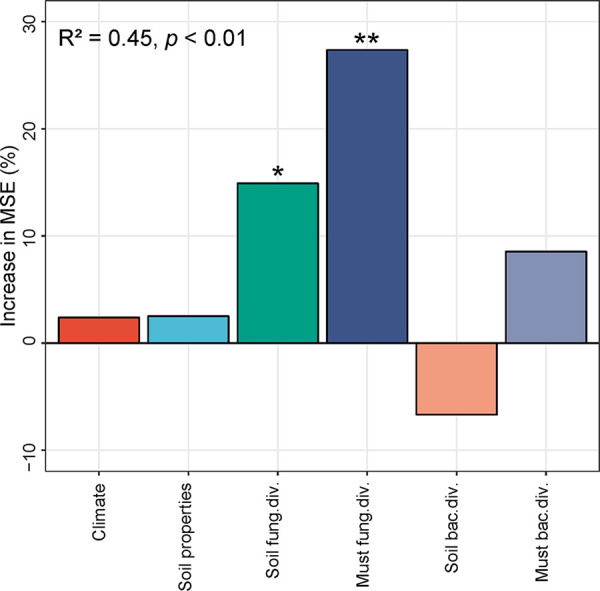
Main predictors of wine regionality. The random forest mean predictor importance is shown by percentage of increase in mean square error (MSE) of climate, soil properties, and microbial diversity (Shannon index) according to wine regionality. Soil bac. div., soil bacterial diversity; Soil fung. div., soil fungal diversity; Must bac. div., must bacterial diversity; Must fung. div., must fungal diversity. Significance levels: *, *P* < 0.05; **, *P* < 0.01.

**FIG 5:**
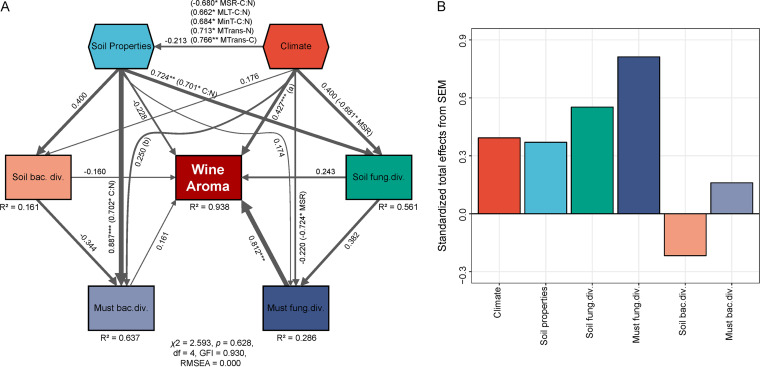
Direct and indirect effects of climate, soil properties, and microbial diversity (Shannon index) on wine regionality. (A and B) Structural equation modeling (SEM) fitted to the diversity of wine aroma profiles (A) and standardized total effects (direct plus indirect effects) derived from the model (B). Climate and soil properties represent composite variables encompassing multiple observed parameters (see Materials and Methods for the complete list of factors used to generate this model). Numbers adjacent to arrows are path coefficients and indicative of the effect size of the relationship. The width of arrows is proportional to the strength of path coefficients. *R*^2^ denotes the proportion of variance explained. (A) (0.747* MT) (0.666* MLT) (0.686* MinT) (−0.875** MSR). (B) (0.753* MinT) (0.772* MLT) (−0.683* MSR) (−0.737* MHT) (−0.843** MaxT). C, soil carbon; N, soil nitrogen; C:N, soil carbon/nitrogen ratio; MSR, mean solar radiation; MT, mean temperature; MLT, mean low temperature; MHT, mean high temperature; MinT, minimum temperature; MaxT, maximum temperature; MTrans, mean transpiration. Significance levels: *, *P* < 0.05; **, *P* < 0.01; ***, *P* < 0.001.

Alongside regional patterns in soil and must microbiota, environmental measures of the wine-growing regions displayed significant differences, such as in C and N levels in soil, solar radiation, and temperature and weather/climatic conditions during the growing season (October 2017–April 2018) (see Table S5 for a complete list). To disentangle the roles of microbial ecology in wine regionality, we used random forest modeling (37) to identify the biotic predictors (soil and must microbial diversity) and abiotic predictors (soil and weather parameters) structuring wine regionality and used structural equation modeling (SEM) (38) to test whether the relationship between microbial diversity and wine regionality would be able to be maintained while accounting for multiple factors simultaneously. The random forest model (*R*^2^ = 0.451, *P* < 0.01) demonstrated that fungal diversity was a predictor for wine regionality. Not surprisingly, must fungal diversity showed higher importance on the model (increase in the mean square error [MSE]) than soil (Fig. 4). The SEM explained 93.8% of the variance found in the pattern of wine regionality (Fig. 5A). Weather correlated with wine aroma profiles directly (especially MT [mean temperature], MLT [mean low temperature], MinT [minimum temperature], and MSR [mean solar radiation]) and indirectly by powerful effects on soil and must microbial diversity, in particular, showing strong effects on soil fungal diversity (Fig. 5A). Must fungal diversity had the highest direct positive effect on wine aroma characteristics, with direct effects by soil fungal diversity (Fig. 5A). Weather, climate, and soil nutrient pools were related primarily through MSR, MLT, MinT, and MTrans (mean transpiration). Soil properties showed strong effects on soil microbial diversity and must bacterial diversity but weak effects on must fungal diversity (Fig. 5A). Must bacterial diversity had a weak effect on wine aroma profiles, as did soil bacterial diversity. Overall, must fungal diversity was the most important predictor of wine characteristics, followed by soil fungal diversity, as indicated by the standardized total effects from SEM (Fig. 5B), with effects from weather and soil properties operating both directly and indirectly (Fig. 5A).

10.1128/mSphere.00534-20.10 TABLE S5 ANOVA results representing soil properties and weather conditions among wine-growing regions in 2017. Download Table S5, XLSX file, 0.01 MB. Copyright © 2020 Liu et al. 2020 Liu et al. This content is distributed under the terms of the Creative Commons Attribution 4.0 International license.

### Source tracking of wine-related fungi within vineyard.

**FIG 6:**
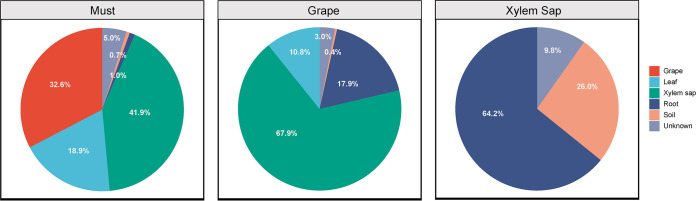
Fungal communities in musts emerge from multiple sources in the vineyard but primarily from grapes and xylem sap. Percent composition representing the contributions of possible sources from the vineyard to the wine-related fungal community are given for must, grape, and xylem sap.

As shown in the SEM, must fungal diversity was correlated with soil fungal diversity, and the former had higher effects on wine aroma profiles (Fig. 5). Given that soil is a potential source of fungi associated with wine production (14), here, we attempt to uncover the mechanism whereby soil fungi are transported from soil to the grapes. We sampled fungal communities from grapevines and soil and hypothesized that the xylem/phloem was the internal mechanism to transport microbes. A total of 2,140,820 ITS high-quality sequences were generated from soil and grapevine samples (grape, leaf, xylem sap, root), which were clustered into 4,050 fungal OTUs with 97% pairwise identity. Using SourceTracker (39), fungal communities in the must were matched to multiple sources from below the ground to above the ground. Results showed that grape and xylem sap were primary sources of must fungi, with 32.6% and 41.9% contributions, respectively (Fig. 6). The fungal structure of xylem sap was similar to that seen with must (Fig. S4A). Further source tracking revealed that the root and soil contributed 90.2% of fungal OTUs of xylem sap and that the latter contributed 67.9% of the fungi of grapes (Fig. 6).

10.1128/mSphere.00534-20.5 FIG S4 Xylem sap is a medium potentially usable for translocation of yeasts from the soil to the grape. (A) Fungal taxa of xylem sap from the vineyard at the genus level. (B) Relative abundances of S. cerevisiae in soil, root, xylem sap, grape, and must. (C and D) Yeast species cultured from xylem sap from vineyard (C) and glasshouse (D). (E to G) Nutrient compositions of xylem sap: carbohydrates (E), total amino acids (protein and free) (F), and organic acids (G). Download FIG S4, TIF file, 2.0 MB. Copyright © 2020 Liu et al. 2020 Liu et al. This content is distributed under the terms of the Creative Commons Attribution 4.0 International license.

Notably, S. cerevisiae yeasts were found shared between microhabitats of soil, root, xylem sap, grape, and must, with the highest (1.22%) and lowest (0.038%) relative abundances in the root and soil, respectively (Fig. S4B). Could xylem vessels represent a translocation pathway of S. cerevisiae from roots to the aboveground? Chemical analysis of nutrient compositions showed that xylem sap contained nine carbohydrates (predominantly glucose, fructose, and sucrose), 15 amino acids (mainly arginine, aspartic acid, and glutamic acid), and six organic acids (primarily oxalic acid), which could be utilized as carbon and nitrogen sources and support yeast growth (Fig. S4E to G) (40). However, no S. cerevisiae yeasts were isolated; distinct isolates of the *Basidiomycota* yeasts of *Cryptococcus* spp. (primarily C. saitoi) and Rhodotorula slooffiae were found instead (Fig. S4C). The data indicating the exclusive existence of these species were validated by isolation from xylem/phloem sap coming from grapevines grown in the glasshouse (Fig. S4D).

## DISCUSSION

Microbial ecology can influence grapevine health and growth, fermentation, flavor characteristics, and wine quality and style (13, 14, 21). We systematically investigated the microbiome from the soil to wine and found that soil and grape must microbiota exhibited regional patterns and that these patterns correlated with resulting wine metabolites. Here, we show that wine regionality is closely associated with fungal ecology, with effects from local weather, climate, and soil properties. A new mechanism to transfer fungi from the soil to grapes and must via xylem sap was investigated.

### A microbial component of wine *terroir*.

Regional spatial patterns have been proposed for soil and grape must microbiota (13, 26–28, 41). The most abundant bacterial phyla in the vineyard soils in our study were *Actinobacteria*, *Proteobacteria*, and *Acidobacteria*, which are known to be dominant and ubiquitous in vineyard soil (24, 26, 27, 42). Among fungi, we recovered 14 phyla, 30 classes, 65 orders, 125 families, and 216 genera, recording a higher diversity than reported in other wine-producing areas in the world (27, 28, 43). *Glomeromycota*, the phylum of arbuscular mycorrhizal fungi reported to positively affect grapevine growth, was reported as abundant in New Zealand vineyards (43). In our study, which analyzed amplicon sequences at the ITS region (rather than the D1/D2 region analyzed in the study cited in reference 43), *Glomeromycota* was recovered with only low frequency from Mornington and Macedon Ranges vineyards. Clearly, there are differences based on the barcoding region but geographic location may also affect distribution (44), as Coller et al. (2019), using the same ITS1F/2 primers as in our study, retrieved *Glomeromycota* as a core phylum member from vineyards in Italy (27). In the must, both principal fermentation drivers (S. cerevisiae and LAB) and grapevine-associated species (such as *Enterobacteriales* and *Aureobasidium*, which may not be active in the fermentation but possibly interact with plants) were present in different abundances among regions (Fig. 3A and B). The order *Lactobacillales*, representing LAB, was present at an abundance of 0.4% across regions, compared to 29.7% found in California in the United States (13) and 14% in Catalonia in Spain (32).

Environmental factors (such as weather and climate) and geographic features structure microbial diversity and biogeography across various habitats in the soil and plant ecosystems (13, 26, 45–47). In this study, we demonstrated that microbial biogeographic communities were distinct in both vineyard soils and grape musts in southern Australia regardless of the growing season/vintage. This aligns with previous studies on wine microbial biogeography and provides further evidence for microbial *terroir* (reviewed by Liu et al. [29]) (13, 15, 19, 26). Soil bacteria can be used to distinguish wine-growing regions, with impacts from soil properties (Fig. 5A), and this is supported by previous work in this field (24, 26, 41). An interesting finding was that must bacterial diversity is strongly affected by soil properties, in particular, by carbon-nitrogen (C/N) ratios. Previous work has shown that must and soil community structures are similar and that some *Enterobacteriales* and *Actinomycetales* species originate from the soil (8, 16). As C/N ratios can be manipulated by composting and cover crops (48), there is an opportunity to manipulate wine microbiota by focusing on vineyard management (29). The soil bacterial microflora is recognized as important for plant growth processes more broadly (49), but fungal diversity beyond endosymbiotic mycorrhizae has not been systematically investigated for grapevines. Here, we show that soil fungal communities are distinct between regions. Our modeling suggests that soil properties and weather strongly affect soil fungal diversity, which was in line with large-scale studies in which climatic factors (especially precipitation) and edaphic factors (especially C/N ratios) were found to be the best predictors of soil fungal richness and community composition (47, 50). Must fungal diversity was also found to be affected by weather and soil properties indirectly via soil fungi that had direct effects on wine aroma profiles (Fig. 5). Considering the limitations represented by the sampling size (15 vineyards) and the data from weather stations, numerous geophysical factors and microclimatic conditions within specific vineyards could explain microbial variations beyond the scope of our measurements. Future studies performed with further sampling within regions and more-precise weather and climate data (for example, real-time weather monitoring within the vineyard) will provide further perspectives with respect to wine microbial biogeography and the response of microbes to local environmental conditions.

It is noteworthy that the drivers of microbial patterns change during wine fermentation. Microbial diversity decreases as alcoholic fermentation proceeds, with a clear loss of microorganisms, including filamentous fungi, non-*Saccharomyces* yeasts (for example, M. guilliermondii), spoilage bacteria (*Acidobacteria* and *Proteobacteria*), and other bacteria with unknown fermentative functions (for example, *Chloroflexi*) (Fig. 3C and D), and the biogeographic trend was lost by the stage of MLF-End (see Table S3 in the supplemental material). This trend was observed more distinctly in fungal communities than in bacterial communities (see Fig. S3A and B in the supplemental material). This was not unexpected as it is clear that fermentation affects fungal populations more strongly than bacterial populations, due to increasing fermentation rate, temperature, and ethanol concentration induced by S. cerevisiae growth (11, 51). In this case, fermentation conditions, such as the chemical environment and interactions and/or competition within the community (11, 52), reshape the observed microbial patterns. Despite the complex microbial ecosystem changes occurring during fermentation, we show that biogeographic patterns in the must could be reflected in the regional metabolic profiling of wine. Our modeling indicates that the indirect effects on wine aroma profiles of weather and soil properties via influencing soil and must microbial diversity are more powerful than the direct effects (Fig. 5). In the resulting wines, the most volatile compounds were alcohols, esters, acids, and aldehydes (Table S2), many of which were likely microbial products. Some compounds, for example, monoterpenes, are derived from grapes and are modified by yeast and bacterial metabolism during fermentation (10). These modellings are potentially important to inform farming practices to structure regional microbial communities that can benefit soil quality and thus crop productivity.

### Fungal communities distinguish wine quality and style.

In grape musts, bacterial and fungal communities exhibit different responses to site-specific and environmental effects. Bacterial regional patterns were not as distinct as fungal regional patterns and were significantly impacted by vintage (Table S3). Although they showed profound relationships with soil properties (for example, C/N ratios) and affected wine fermentation, must bacteria exhibited insignificant effects on wine aroma profiles (Fig. 5). In contrast, fungal communities displayed diverse distribution patterns at the regional scale and were weakly or insignificantly impacted by vintage in this study, aligning with results presented by Bokulich et al. (2014) (13). Soil fungal communities are less diverse than bacterial communities (Fig. S2) (44) but are of more importance to the resultant wine regionality (see Fig. 4 and 5). Must fungi, in particular, the fermentative yeasts, participate in alcoholic fermentation processes and provide aroma compounds to structure wine flavor (10). As indicated by SEM, soil fungal communities are affected by local soil properties and weather and exert impacts on must fungal communities (Fig. 5). One explanation is that grapevines filter soil microbial taxa, selecting for grape and must consortia (53, 54). Beyond fungi, plant fitness is linked strongly to the responses of soil microbial communities to environmental conditions (55). More-sensitive responses of vineyard soil fungi might improve grapevine fitness with respect to local environments, thus enhancing the expression of regional characteristics of grapes and wines.

How could yeasts present in the soil be transported to the grape berry? Soil is a source reservoir of grapevine-associated microbiota (Fig. 6), an assertion that is supported by previous publications (15, 24, 41, 56). As well as transporting water and minerals absorbed by roots to the photosynthetic organs, xylem sap is also a microhabitat for microbes that can bear its nutritional environment (40). Here, we investigated xylem sap as a conduit to shape the microbiota in the grape by enrichment of the microbes recruited by roots and transported by xylem sap to the grape berries (24, 33, 49). The isolated yeasts belonged to the *Cryptococcus* and *Rhodotorula* genera, indicating that the xylem sap environment is not sterile and can potentially transport yeasts to the phyllosphere. The endophyte Burkholderia phytofirmans strain PsJN has been shown to colonize grapevine roots from the rhizosphere and spread to inflorescence tissues through the xylem (33, 57). While we were unable to find fermentative yeasts in the sap of grapevines, other yeasts (and/or spores) were present and may also be transported within the grapevine as well as making their way to the phyllosphere through other mechanisms (water splashes, insect vectors). As previous studies showed, fermentative yeasts are persistent in vineyards (58, 59, 82) and might be transported through the vine to the grapes (60). We can thus suggest fungi as a signature corresponding to consistent expression of regionality in wine production.

Our study results suggest microbial contributions to wine aroma and that such contributions are related to the environment in which they are grown. Whether geographically differential microbiota can actually sculpt wine characteristics must be further empirically addressed. Fungi are implicated in the interrelationship of biotic and abiotic elements in vineyard ecosystems and could potentially be transported internally within the grapevine. Climate and soil properties profoundly structure microbial patterns from the soil to the grape must and ultimately affect the wine metabolic profile. We do not yet know how grapevines recruit their microbiome to maximize physiological development and maintain microbial diversity under local conditions. The addition of our study in Australia to support and extend investigations in other wine-growing regions worldwide contributes to a complex picture of environment-plant-microbe interactions in production of wine. Further studies focusing on empirical experiments will be indispensable to improve understanding of how agricultural production affects the ultimate flavor of foods and beverages.

## MATERIALS AND METHODS

### Sample sites and weather parameters.

A total of 15 Vitis vinifera cv. Pinot Noir vineyards were selected in 2017 from among those maintained in Geelong, Mornington Peninsula (Mornington), Macedon Ranges, Yarra Valley, Grampians, and Gippsland in southern Australia, with distances between vineyards ranging from 5 km to 400 km (see Fig. S1 in the supplemental material). All these vineyards are operated under conventional management practices, and the vineyard conditions (altitude, orientation, soil conditions, cover crop) are listed in Table S1 in the supplemental material. In 2018, the sampling from the five vineyards in Mornington Peninsula (all <20 km apart) was repeated to elucidate the influence of sampling year (vintage) on microbial patterns and wine profiles. Each site’s Global Positioning System (GPS) coordinates (longitude, latitude, altitude) were utilized to extract weekly weather data from the data set provided by Australian Water Availability Project (AWAP). Variables were observed by robust topography, resolving analysis methods at a resolution of 0.05° by 0.05° (approximately 5 km by 5 km) (61). Weekly measurements for all vineyards were extracted for mean solar radiation (MSR), mean high temperature (MHT), mean low temperature (MLT), maximum temperature (MaxT), minimum temperature (MinT), mean temperature (MT), precipitation, mean relative soil moisture, mean evaporation (soil plus vegetation), and mean transpiration (MTrans) in growing seasons (October 2016/2017 to April 2017/2018).

10.1128/mSphere.00534-20.2 FIG S1 Map of 15 sampling vineyards from six Pinot Noir wine-producing regions in southern Australia, spanning 400 km (east to west [E-W]). Download FIG S1, TIF file, 0.4 MB. Copyright © 2020 Liu et al. 2020 Liu et al. This content is distributed under the terms of the Creative Commons Attribution 4.0 International license.

10.1128/mSphere.00534-20.6 TABLE S1 Vineyard conditions and number of soil, must and ferment, and plant samples collected in this study. Download Table S1, DOCX file, 0.02 MB. Copyright © 2020 Liu et al. 2020 Liu et al. This content is distributed under the terms of the Creative Commons Attribution 4.0 International license.

### Collection of soil, plant, must, and ferment samples.

In each vineyard, soil samples were collected from three sites covering the top, middle, and bottom of the dominant slope at harvest March to April 2017 (*n* = 45) and 2018 (*n* = 15) at depths of 0 to 15 cm and 30 to 50 cm from the grapevine into the interrow (three subsamples were mixed to form a composite sample at each site) (Table S1A). To further investigate fungal ecology in the vineyard, comprehensive vineyard samples (*n* = 50) were collected from two vineyards 5 km apart in the 2018 vintage (Table S1B). These two vineyards were managed by the same winery, and the viticultural management practices were very similar; for example, grapevines were maintained under vertical shoot positioning trellising systems and the same sprays were applied at the same time of year. Five replicate Pinot Noir vines in each vineyard were selected from the top, middle, and bottom of the dominant slope, covering topological profiles of the vineyard. For each grapevine, the following five different sample types were collected at harvest in March 2018: soil (0 to 15 cm deep, root zone), roots, xylem/phloem sap, leaves, and grapes. Xylem sap (*n* = 10) was collected from the shoots using a centrifugation method under aseptic conditions (62) (Table S1B). Details of xylem sap collection, nutrient composition analysis, and yeast isolation were provided in Text S1 in the supplemental material. Samples were stored in sterile bags, shipped on ice, and stored at −80°C until processing.

10.1128/mSphere.00534-20.1 TEXT S1 Description of materials and methods used for collection and analysis of yeast isolated from xylem sap. Download Text S1, DOCX file, 0.02 MB. Copyright © 2020 Liu et al. 2020 Liu et al. This content is distributed under the terms of the Creative Commons Attribution 4.0 International license.

Longitudinal samples were collected to study microbial communities during fermentation at the following five time points: at the must time point (destemmed, crushed grapes prior to fermentation), at early fermentation (AF, with less than 10% of the sugar fermented), at middle of fermentation (AF-Mid, with around 50% of sugar fermented), at the end of fermentation (AF-End, ∼6°Brix, following pressing but prior to barreling), and at the end of malolactic fermentation (MLF-End, in barrels) (Table S1A). The chemical constituents of the initial musts were similar (Table S1) and were fermented in the respective wineries following similar fermentation protocols of 3 days at a cool temperature (known as cold-soaking) followed by warming the must so that fermentation could commence. Fermentations were conducted without addition of commercial yeasts and bacteria. Two fermentations from Grampians and Mornington did not complete the process and were excluded from analysis, giving wine samples from 13 vineyards in the 2017 vintage. Triplicate subsamples from tanks or barrels (from the top, middle, and bottom) were collected and mixed as composite samples. All samples (*n* = 90) were frozen immediately after sampling in the winery, shipped on ice to the laboratory, and stored at −20°C until processing.

### Soil analysis.

Edaphic factors were analyzed to explore the effects of soil properties on wine-related microbiota and aroma profiles. Soil pH was determined in a 2:5 soil/water suspension. Soil carbon (C), nitrogen (N), nitrate, and ammonium were analyzed by Melbourne Trace Analysis for Chemical, Earth and Environmental Sciences (TrACEES) Soil Node, at the University of Melbourne. Total C and N levels were determined using the classic Dumas method of combustion (63) and a Leco TruMac CN analyzer (Leco Corporation, MI, USA) at a furnace temperature of 1,350°C. Nitrate and ammonium were extracted with 2 M KCl and their levels determined on a segmented flow analyzer (SAN++; Skalar, Breda, Netherlands) (63).

### Wine volatile analysis.

To represent the wine aroma, volatile compounds of MLF-End samples were determined using headspace solid-phase microextraction gas-chromatographic mass-spectrometry (HS-SPME–GC-MS) (64, 65) with some modifications. Analyses were performed with an Agilent 6850 GC system and a 5973 mass detector (Agilent Technologies, Santa Clara, CA, USA) equipped with a PAL RSI 120 autosampler (CTC Analytics AG, Switzerland). Briefly, 10 ml wine was added to a 20-ml glass vial with 2 g of sodium chloride and 20 μl of internal standard (4-Octanol; 100 mg/liter) and then equilibrated at 35°C for 15 min. A polydimethylsiloxane/divinylbenzene (PDMS/DVB; Supelco) 65-μm-pore-size SPME fiber was immersed in the headspace for 10 min at 35°C with agitation. The fiber was desorbed in the GC injector for 4 min at 220°C. Volatiles were separated on an Agilent J&W DB-Wax Ultra Inert capillary GC column (30 m by 0.25 mm by 0.25 μm) with helium carrier gas used at a flow rate of 0.7 ml/min. The column temperature program was as follows: holding at 40°C for 10 min, increasing at 3.0°C/min to 220°C, and holding at that temperature for 10 min. The temperature of the transfer line of GC and MS was set at 240°C. The ion source temperature was 230°C. The MS was operated in positive electron ionization (EI) mode with scanning over a mass acquisition range of 35 to 350 *m*/*z*. Raw data were analyzed with Agilent ChemStation software for qualification and quantification (66). Volatile compounds (*n* = 88) were identified in wine samples according to retention indices, reference standards, and mass spectra matching performed with the NIST11 library database. A total of 13 successive levels of standard solution in model wine solutions (12% [vol/vol] ethanol saturated with potassium hydrogen tartrate and adjusted to pH 3.5 using 40% [wt/vol] tartaric acid) were analyzed by the same protocol as was used for the wine samples to establish the calibration curves for quantification. Peak areas of volatile compounds were integrated via a target ion model. The concentrations of volatile compounds were calculated with the calibration curves and used for downstream data analysis.

### DNA extraction and sequencing.

Genomic DNA was extracted from plant and soil samples using PowerSoil DNA isolation kits (Qiagen, CA, USA). DNA extraction from soil and xylem sap followed the kit’s protocols. Wine fermentation samples were thawed, and biomass was recovered by centrifugation at 4,000 × *g* for 15 min, washed three times in ice-cold phosphate-buffered saline (PBS)–1% polyvinylpolypyrrolidone (PVPP), and centrifuged at 10,000 × *g* for 10 min (12). The obtained pellets were used for DNA extraction following the kit protocol. For the grapevine samples, roots, leaves, and grapes (removed seeds and stems) were ground into powder under the protection of liquid nitrogen with 1% PVPP and DNA was isolated afterward following the kit protocol. DNA extracts were stored at −20°C until further analysis.

Genomic DNA was submitted to the Australian Genome Research Facility (AGRF) for amplification and sequencing. To assess the bacterial and fungal communities, the 16S rRNA gene V3-V4 region and partial fungal internal transcribed spacer (ITS) region were amplified using universal primer pairs 341F/806R (67) and ITS1F/2 (68), respectively. The primary PCRs contained 10 ng DNA template, 2× AmpliTaq Gold 360 master mix (Life Technologies, Australia), and 5 pmol of each primer. A secondary PCR was performed with TaKaRa *Taq* DNA polymerase (Clontech) to index the amplicons. Amplification were conducted under the following conditions: for bacteria, 95°C for 7 min, followed by 30 cycles of 94°C for 30 s, 50°C for 60 s, and 72°C for 60 s and a final extension at 72°C for 7 min; for fungi, 95°C for 7 min, followed by 35 cycles of 94°C for 30 s, 55°C for 45 s, and 72°C for 60 s and a final extension at 72°C for 7 min. PCR products were purified, quantified, and pooled at the same concentration (5 nM). The resulting amplicons were cleaned again using magnetic beads, quantified by fluorometry (Promega QuantiFluor), and normalized. The equimolar pool was cleaned a final time using magnetic beads to concentrate the pool and then measured using a D1000 high-sensitivity tape on an Agilent 2200 TapeStation. The pool was diluted to 5 nM, and molarity was confirmed again using a D1000 high-sensitivity tape. This was followed by 300-bp paired-end sequencing performed on an Illumina MiSeq system (San Diego, CA, USA).

Raw sequences were processed using QIIME v1.9.2 (69). Low-quality regions (Q < 20) were trimmed from the 5′ end of the sequences, and the paired ends were joined using FLASH (70). Primers were trimmed and a further round of quality control was conducted to discard full-length duplicate sequences, short (<100-nt) sequences, and sequences with ambiguous bases. Sequences were clustered followed by chimera checking using UCHIME algorithm from USEARCH v7.1.1090 (71). Operational taxonomic units (OTUs) were assigned using a UCLUST open-reference OTU-picking workflow with a threshold of 97% pairwise identity (71). Singletons or unique reads in the resultant data set were discarded; in addition, chloroplast-related and mitochondrion-related reads were removed from the OTU data set for 16S rRNA. Taxonomy was assigned to OTUs in QIIME using the Ribosomal Database Project (RDP) classifier (72) against the GreenGenes bacterial 16S rRNA database (v13.8) (73) for bacteria or the UNITE fungal ITS database (v7.2) (74) for fungi. To avoid/reduce biases generated by the use of various sequencing depths, sequence data were rarefied to the same depth per sample (the lowest sequencing depth of each batch, that is, for the soil, must and wine, and soil and plant samples) prior to downstream analysis.

### Data analysis.

Microbial alpha-diversity was calculated using the Shannon index (*H*) in R (v3.5.0) with the “vegan” package (75). One-way analysis of variance (ANOVA) was used to determine whether sample classifications (e.g., region, fermentation stage) contained statistically significant differences in diversity. Principal-coordinate analysis (PCoA) was performed to evaluate the distribution patterns of wine metabolome and wine-related microbiome based on beta-diversity calculated by Bray-Curtis distance determinations performed with the “labdsv” package (76). Permutational multivariate analysis of variance (PERMANOVA) was conducted within each sample classification using distance matrices with 999 permutations to determine statistically significant differences by the use of the “adonis” function in “vegan” (75).

Significant differences of wine microbiome between fermentation stages were tested based on taxonomic classification using linear discriminant analysis (LDA) effect size (LEfSe) analysis (77) (https://huttenhower.sph.harvard.edu/galaxy/). The OTU table was filtered to include only OTUs with >0.01% relative abundance to reduce LEfSe complexity. This method applies the factorial Kruskal-Wallis rank sum test (α = 0.05) to identify taxa with significant differential abundances between categories (using all-against-all comparisons), followed by the logarithmic LDA score (threshold = 2.0) to estimate the effect size of each discriminative feature. Significant taxa were used to generate taxonomic cladograms illustrating differences between sample classes.

A random forest supervised-classification model (37) was employed to identify the main predictors of wine regionality among the following variables: must and soil microbial diversity (Shannon index), soil properties, and weather. The importance of each predictor was determined by evaluating the decrease in prediction accuracy (that is, the increase in the mean square error [MSE] corresponding to comparisons between observations and out-of-bag predictions) when the data were randomly permuted for the predictor. This analysis was conducted with 5,000 trees using the “randomForest” package in R (78). The significance of the model (*P* values) and the leave-one-out cross-validation *R*^2^ values were assessed using the “A3” package (ntree = 5,000) (79). Structural equation modeling (SEM) (38) was used to evaluate the direct and indirect relationships among must and soil microbial diversity, soil properties, climate, and wine regionality. SEM is an *a priori* approach partitioning the influences of multiple drivers in a system to help characterize and comprehend complex networks of ecological interactions (80). An *a priori* model was established based on the known effects and relationships among these drivers of regional distribution patterns of wine aroma to manipulate the data before modeling. Weather and soil properties were used as composite variables (both random forest and SEM) to collapse the effects of multiple conceptually related variables into a single composite effect, thus aiding interpretation of model results (38). A path coefficient describes the strength and sign of the relationship between two variables (38). The good fit of the model was validated by the χ^2^ test (*P* *>* 0.05), using the goodness-of-fit index (GFI > 0.90) and the root MSE of approximation (RMSEA < 0.05) (81). The standardized total effects of each factor on the wine regionality pattern were calculated by summing all direct and indirect pathways between two variables (38). All the SEM analyses were conducted using AMOS v25.0 (IBM, NY, USA).

SourceTracker was used to track potential sources of wine-related fungi within the vineyards (39). SourceTracker represents a Bayesian approach that treats each give community (sink) as a mixture of communities deposited from a set of source environments and estimates the proportion of taxa in the sink community that come from possible source environments. When a sink contains a mixture of taxa that do not match any of the source environments, that portion of the community is assigned to an “unknown” source (39). In this model, we examined musts (*n* = 2) and vineyard sources (*n* = 50), including grapes, leaves, xylem sap, roots, and soils. The OTU tables were used as data input for modeling using the “SourceTracker” R package (https://github.com/danknights/sourcetracker).

### Data availability.

Raw data are publicly available in the National Centre for Biotechnology Information Sequence Read Archive under BioProject accession numbers PRJNA594458 (bacterial 16S rRNA sequences) and PRJNA594469 (fungal ITS sequences).
