# Supplementary figures and images for: The Fungal Microbiome Is an Important Component of Vineyard Ecosystems and Correlates with Regional Distinctiveness of Wine
Source: mSphere. 2020 Aug 12;5(4):e00534-20. doi: 10.1128/mSphere.00534-20 (PMC7426168; doi:10.1128/mSphere.00534-20)

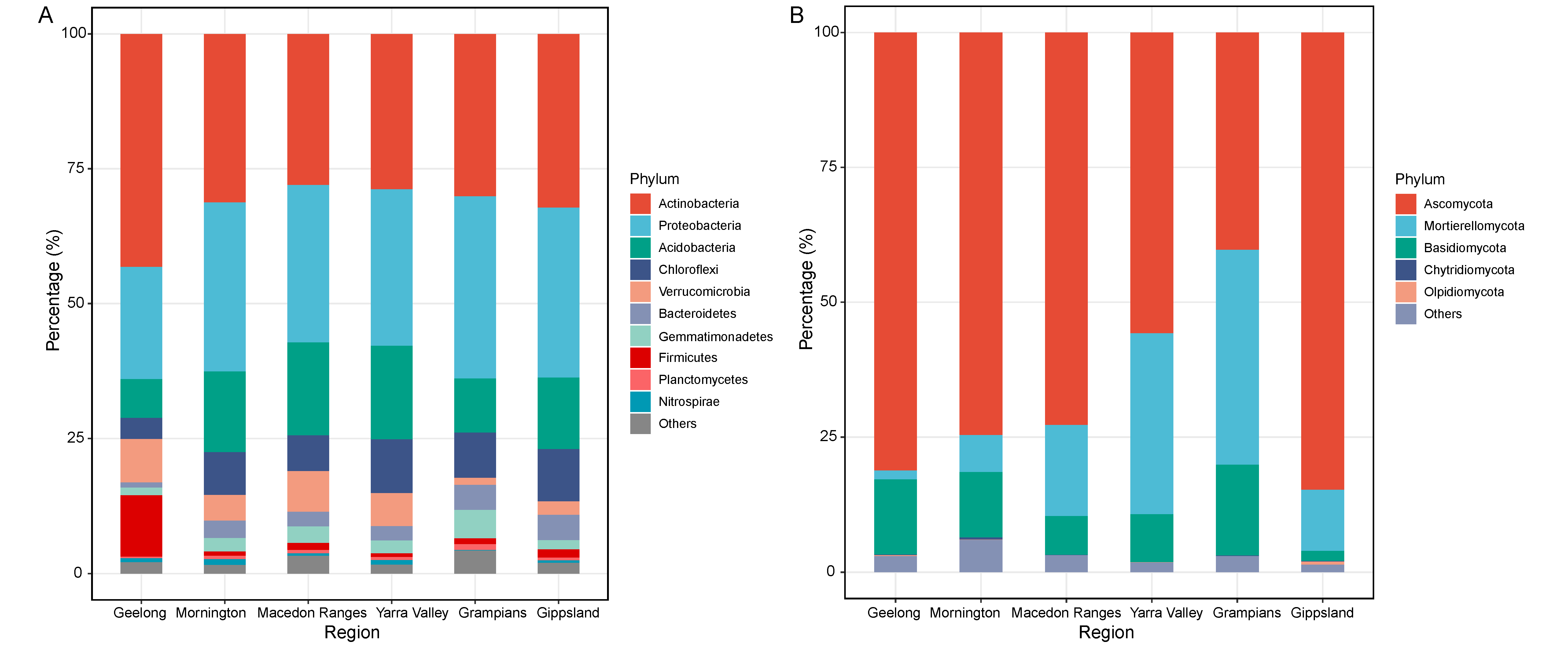

Supplement: FIG S2 [file mSphere.00534-20-sf002.tif]

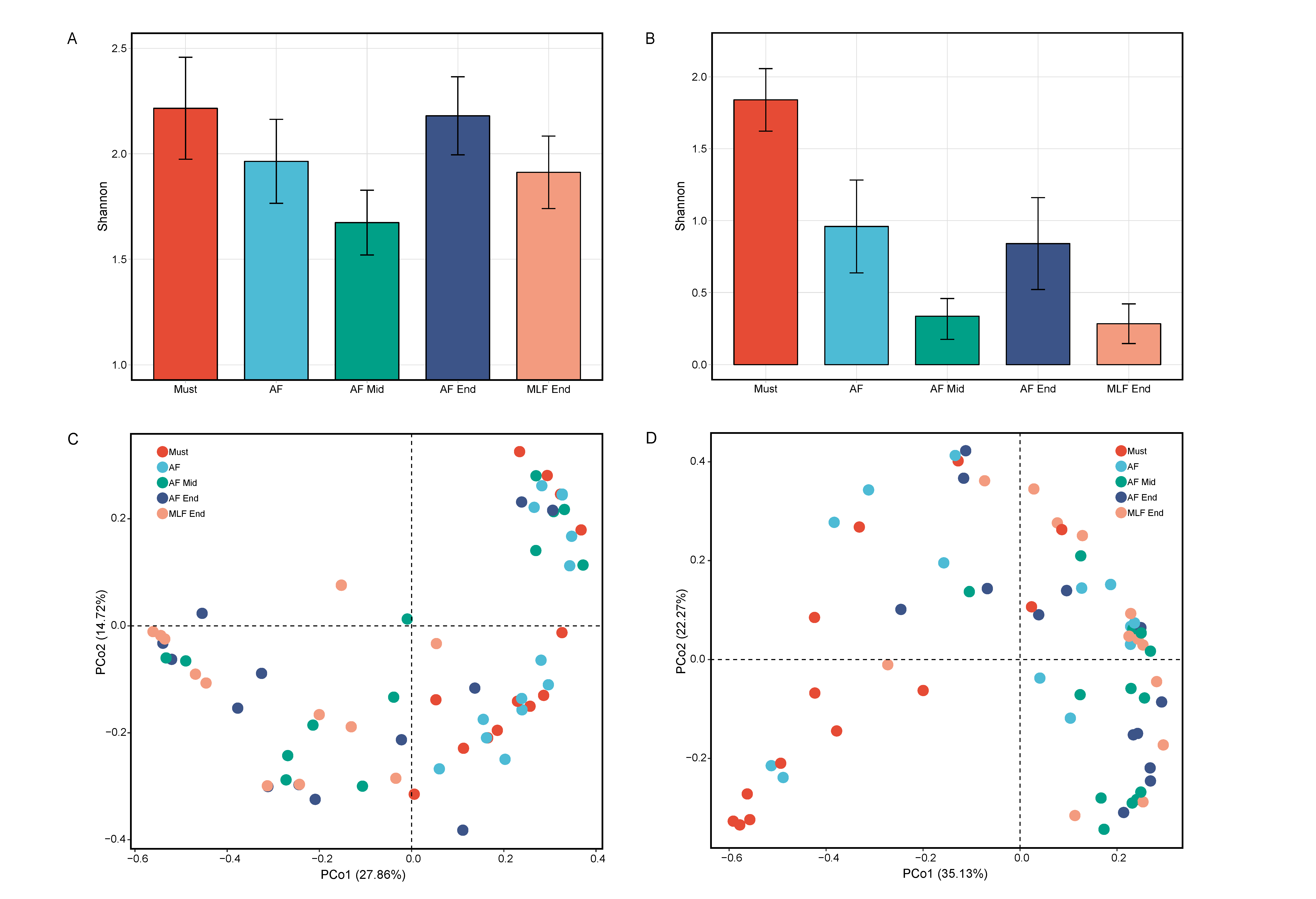

Supplement: FIG S3 [file mSphere.00534-20-sf003.tif]

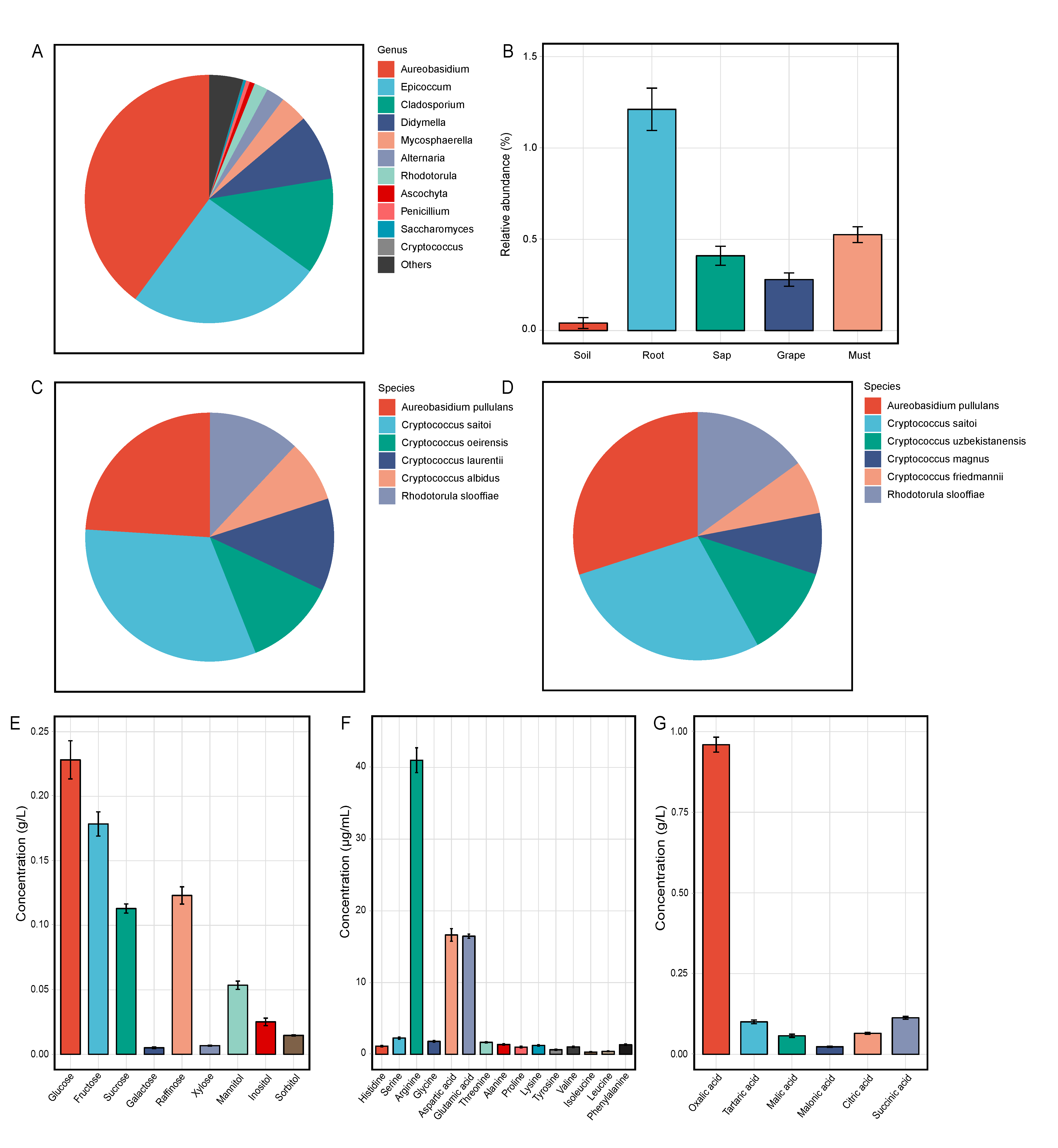

Supplement: FIG S4 [file mSphere.00534-20-sf004.tif]

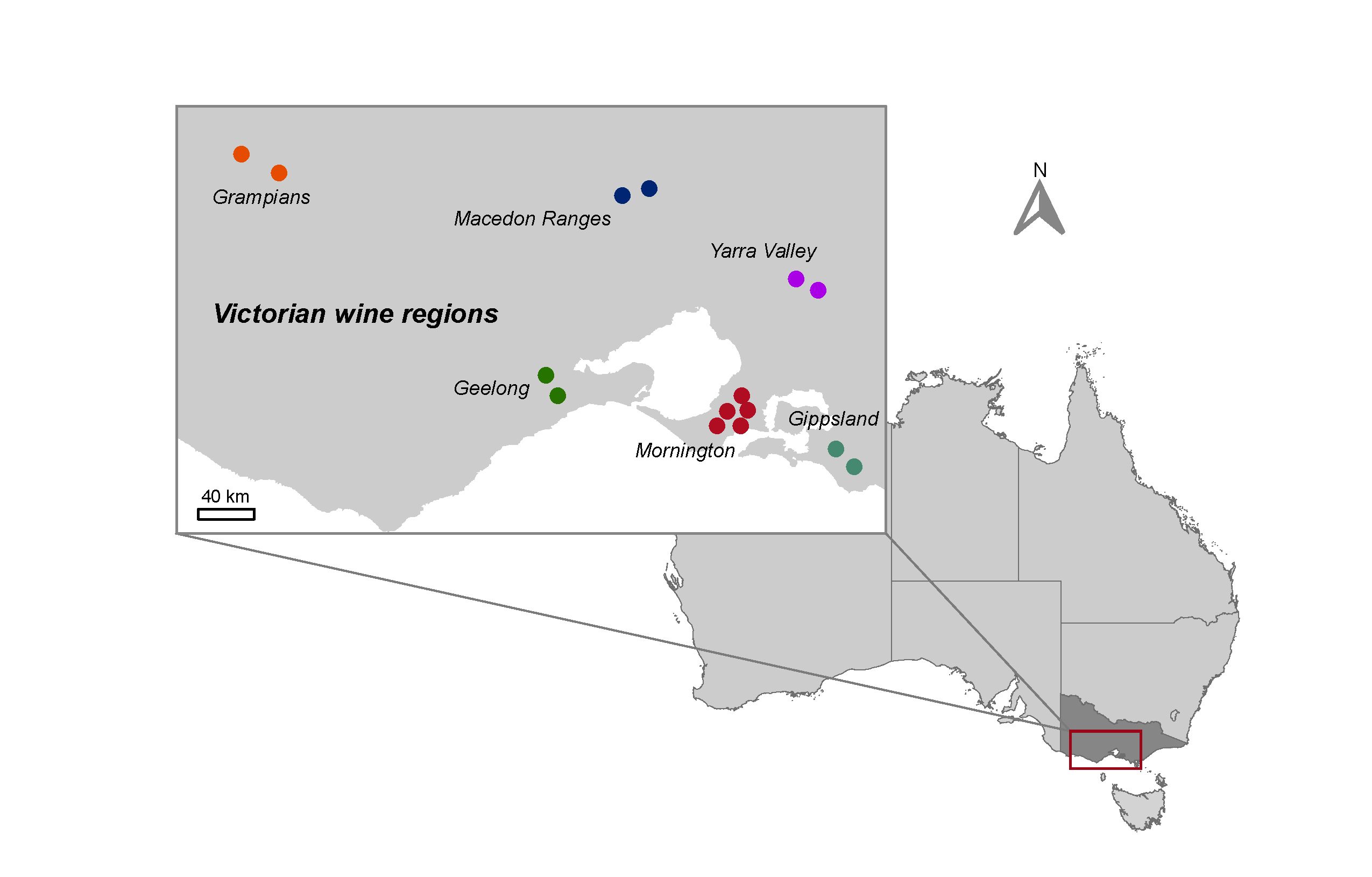

Supplement: FIG S1 [file mSphere.00534-20-sf001.tif]
