# Supplementary material for: The Fungal Microbiome Is an Important Component of Vineyard Ecosystems and Correlates with Regional Distinctiveness of Wine
Source: mSphere. 2020 Aug 12;5(4):e00534-20. doi: 10.1128/mSphere.00534-20 (PMC7426168; doi:10.1128/mSphere.00534-20)
Supplement: TABLE S1 [file mSphere.00534-20-st001.docx]

# **Supplementary Tables**

**Table S1 Vineyard conditions and number of soil, must and ferments, and plant samples collected in this study**

| (A) Soil, must and ferment samples | | |  |  |  |  |  |  |  |  |  |  |  |  |  |
| --- | --- | --- | --- | --- | --- | --- | --- | --- | --- | --- | --- | --- | --- | --- | --- |
| Vintage | Region | Vineyard (V) | Altitude | Orientation | Soil subgroup | Soil texture | Cover crop | Chemical constituents of harvested grapes | | Sample Fraction | | | | | |
|  |  |  |  |  |  |  |  | °Brix | pH | Soil | Must^1^ | AF^1^ | AF-Mid^1^ | AF-End^1^ | MLF-End^1,2^ |
| 2017 | Geelong | V1 | 82 | N | Sodosols | Clay loam | Grass | 19.25 | 3.68 | 3 | 1 | 1 | 1 | 1 | 1 |
|  |  | V2 | 92 | N | Sodosols | Clay loam | Grass | 19.82 | 3.64 | 3 | 1 | 1 | 1 | 1 | 1 |
|  | Mornington | V3 | 101 | N | Sodosols | Light medium clay | Grass | 19.67 | 3.62 | 3 | 1 | 1 | 1 | 1 | 1 |
|  |  | V4 | 135 | N | Sodosols | Heavy clay | Grass | 19.65 | 3.57 | 3 | 1 | 1 | 1 | 1 | 1 |
|  |  | V5 | 223 | N | Ferrosols | Clay loam | Grass | 19.26 | 3.72 | 3 | 1 | 1 | 1 | 1 | 1 |
|  |  | V6 | 195 | N | Ferrosols | Heavy clay | Grass | 19.15 | 3.56 | 3 | 1 | 1 | 1 | 1 | 1 |
|  |  | V7 | 55 | N | Sodosols | Heavy clay | Grass | 18.53 | 3.65 | 3 | N/A | N/A | N/A | N/A | N/A |
|  | Macedon Ranges | V8 | 614 | NE | Chromosols | Light medium clay | Grass | 18.30 | 3.48 | 3 | 1 | 1 | 1 | 1 | 1 |
|  |  | V9 | 515 | N | Ferrosols | Light medium clay | Grass | 17.90 | 3.46 | 3 | 1 | 1 | 1 | 1 | 1 |
|  | Yarra Valley | V10 | 119 | N | Chromosols | Heavy clay | Grass | 19.52 | 3.68 | 3 | 1 | 1 | 1 | 1 | 1 |
|  |  | V11 | 174 | N | Chromosols | Heavy clay | Grass | 19.86 | 3.64 | 3 | 1 | 1 | 1 | 1 | 1 |
|  | Grampians | V12 | 344 | N | Sodosols | Clay loam | Grass | 19.75 | 3.58 | 3 | 1 | 1 | 1 | 1 | 1 |
|  |  | V13 | 356 | N | Sodosols | Clay loam | Grass | 19.52 | 3.62 | 3 | N/A | N/A | N/A | N/A | N/A |
|  | Gippsland | V14 | 38 | NE | Dermosols | Clay loam | Grass | 18.50 | 3.68 | 3 | 1 | 1 | 1 | 1 | 1 |
|  |  | V15 | 46 | N | Dermosols | Clay loam | Grass | 18.81 | 3.62 | 3 | 1 | 1 | 1 | 1 | 1 |
| 2018 | Mornington | V3 | 101 | N | Sodosols | Light medium clay | Grass | 20.32 | 3.43 | 3 | 1 | 1 | 1 | 1 | 1 |
|  |  | V4 | 135 | N | Sodosols | Heavy clay | Grass | 19.85 | 3.61 | 3 | 1 | 1 | 1 | 1 | 1 |
|  |  | V5 | 223 | N | Ferrosols | Clay loam | Grass | 20.61 | 3.48 | 3 | 1 | 1 | 1 | 1 | 1 |
|  |  | V6 | 195 | N | Ferrosols | Heavy clay | Grass | 19.70 | 3.65 | 3 | 1 | 1 | 1 | 1 | 1 |
|  |  | V7 | 55 | N | Sodosols | Heavy clay | Grass | 19.54 | 3.52 | 3 | 1 | 1 | 1 | 1 | 1 |
| ^1^ For microbial analysis: triplicates from tanks/ barrels were collected and mixed as one composite sample which was analysed by NGS | | | | | | | | |  |  |  |  |  |  |  |
| ^2^ For wine aroma analysis: triplicates were analysed by GC-MS | | | |  |  |  |  |  |  |  |  |  |  |  |  |
| N/A: not applied, spontaneous fermentations did not complete and were excluded from analysis | | | | | |  |  |  |  |  |  |  |  |  |  |

| (B) Soil and plant samples | |  |  |  |  |  |  |  |
| --- | --- | --- | --- | --- | --- | --- | --- | --- |
| Vintage | Region | Vineyard (V) | Vine (v) | Sample Fraction | | | | |
|  |  |  |  | Soil | Root | Xylem sap | Leaf | Grape |
| 2018 | Mornington | V5 | v1 | 1 | 1 | 1 | 1 | 1 |
|  |  |  | v2 | 1 | 1 | 1 | 1 | 1 |
|  |  |  | v3 | 1 | 1 | 1 | 1 | 1 |
|  |  |  | v4 | 1 | 1 | 1 | 1 | 1 |
|  |  |  | v5 | 1 | 1 | 1 | 1 | 1 |
