# Supplementary material for: The Fungal Microbiome Is an Important Component of Vineyard Ecosystems and Correlates with Regional Distinctiveness of Wine
Source: mSphere. 2020 Aug 12;5(4):e00534-20. doi: 10.1128/mSphere.00534-20 (PMC7426168; doi:10.1128/mSphere.00534-20)
Supplement: TEXT S1 [file mSphere.00534-20-s0001.docx]

# **Supplementary Materials and Methods**

**Xylem sap collection**

Vine shoots (n = 10) were harvested from the vineyard (Table S1) under aseptic conditions and then centrifuged (1). The shoots were peeled to remove the outer bark and phloem layer, sterilised with 70% ethanol and cut to fit into 10-ml sterile centrifuge tubes that had 10 sterile glass beads at the bottom. The shoots were centrifuged at 15,000 *× g* for 10 min at 4°C and the xylem sap collected giving ~ 2.0 mL per sample. Additional xylem sap (n = 5) were sampled from *Vitis vinifera* Shiraz grapevines grown in a glasshouse at the University of Melbourne. Xylem fluid was extracted with a pressure cylinder apparatus (similar to a Scholander pressure chamber) (2). Grapevines were uprooted and the soil was carefully removed from the roots. On an aseptic bench, the main trunk was cut 5-10 cm above the roots with a sterile blade, girdled to remove phloem tissue, sterilised by immersion in 70% ethanol, and immediately inserted into a pressure cylinder. The cylinder applied 60-70 kPa pressure for two hours to extract the xylem sap (~ 4.0 mL). Xylem sap was divided into two subsamples, one used immediately to isolate living yeasts and the other flash frozen with liquid nitrogen and stored at -80°C for DNA extraction, next-generation sequencing and chemical analysis.

**Chemical analysis of xylem sap composition**

Carbohydrates of xylem sap were determined using enzymatic methods by Megazyme assay kits (Megazyme, Ireland) following the manufacturer’s protocol. Amino acids (free and protein- bound) were determined using pre-column derivatisation with 6-aminoquinolyl-N-hydroxysuccinimidyl carbamate followed by separation and quantification with the ACQUITY Ultra Performance LC (UPLC; Waters, MA, USA) system at the Australian Proteome Analysis Facility. The column was an ACQUITY UPLC BEH C18 column (1.7 μm × 2.1 mm × 5 mm) with detection at 260 nm (UV) and a flow rate of 0.7 mL/min at 57–60°C. Identification and quantitation of the amino acids was performed against a set of prepared standards, with DL-norvaline as the internal standard (3, 4). Organic acids were determined using a Waters High-performance liquid chromatography (HPLC) (Waters, MA, USA) based on Andersen et al (1989) (5) with modification. Here, 20 μL xylem sap was injected through a Synergi™ Hydro-RP LC Column (250 mm × 4.6 mm × 4 μm; Phenomenex Inc, CA, USA) at 60°C, with detection at 210 nm (UV). Mobile phases at a flow rate of 1.0 mL/min, with 20 mM potassium phosphate buffer (A, pH = 1.5) and 100% methanol (B) following a gradient programme: (0-2.5) min, 100% A; (2.5-2.9) min, linear ramp to 30% B; (2.9-8.0) min, 30% B; (8.0-8.5) min, linear ramp to 100% A; (8.5-10) min, 100% A. Identification and quantitation of the compounds was performed using a set of prepared standards.

**Isolation and identification of yeasts from xylem sap**

Yeasts were isolated and identified from xylem sap to explore the potential translocation mechanism of yeasts in the vineyard. Xylem sap was serially diluted and plated (0.1 mL) onto solid yeast extract peptone dextrose (YPD) medium that was supplemented with 34 mg/mL chloramphenicol and 25 mg/mL ampicillin to inhibit bacterial growth. Plates were incubated at 28°C for 2-3 days in aerobic conditions. Single colonies with different morphological types were streaked onto Wallerstein Nutrient (WLN) agar media to obtain pure cultures. DNA was recovered from pure colonies using the MasterPure™ Yeast DNA Purification Kit (Epicentre, Madison, WI) following the manufacturer’s instructions. The 26S rDNA D1/D2 domain was amplified using primers NL1/4 (6) for sequencing by Australian Genome Research Facility (AGRF). Sequences were trimmed, aligned and analysed using BLAST at NCBI (<http://blast.ncbi.nlm.nih.gov/Blast.cgi>). Sequence data was uploaded to Genbank with accession numbers MN847682 - MN847692.

**References:**

1. Jones DA, Wang W, Fawcett R. 2009. High-quality spatial climate data-sets for Australia. Australian Meteorological and Oceanographic Journal 58:233.

2. Schurr U. 1998. Xylem sap sampling—new approaches to an old topic. Trends in Plant Science 3:293-298.

3. Cohen SA. 2001. Amino acid analysis using precolumn derivatization with 6-aminoquinolyl-N-hydroxysuccinimidyl carbamate, p 39-47, Amino Acid Analysis Protocols. Springer.

4. Cohen SA, Michaud DP. 1993. Synthesis of a fluorescent derivatizing reagent, 6-aminoquinolyl-N-hydroxysuccinimidyl carbamate, and its application for the analysis of hydrolysate amino acids via high-performance liquid chromatography. Analytical biochemistry 211:279-287.

5. Andersen PC, Brodbeck BV, Mizell RF. 1989. Metabolism of amino acids, organic acids and sugars extracted from the xylem fluid of four host plants by adult Homalodisca coagulata. Entomologia Experimentalis et Applicata 50:149-159.

6. Kurtzman CP, Robnett CJ. 1998. Identification and phylogeny of ascomycetous yeasts from analysis of nuclear large subunit (26S) ribosomal DNA partial sequences. Antonie van Leeuwenhoek 73:331-371.
